# Why Are Bifidobacteria Important for Infants?

**DOI:** 10.3390/microorganisms10020278

**Published:** 2022-01-25

**Authors:** Gerrit A. Stuivenberg, Jeremy P. Burton, Peter A. Bron, Gregor Reid

**Affiliations:** 1 Centre for Human Microbiome and Probiotic Research, Lawson Health Research Institute, London, ON N6A4V2, Canada; gstuiven@uwo.ca (G.A.S.); Jeremy.Burton@LawsonResearch.Com (J.P.B.); 2 Departments of Microbiology and Immunology and Surgery, Western University, London, ON N6A 3K7, Canada; 3 Seed Health Inc., Venice, CA 90291, USA; peter.bron@seed.com

**Keywords:** *Bifidobacterium*, infants, gut microbiome, probiotics

## Abstract

The presence of *Bifidobacterium* species in the maternal vaginal and fecal microbiota is arguably an evolutionary trait that allows these organisms to be primary colonizers of the newborn intestinal tract. Their ability to utilize human milk oligosaccharides fosters their establishment as core health-promoting organisms throughout life. A reduction in their abundance in infants has been shown to increase the prevalence of obesity, diabetes, metabolic disorder, and all-cause mortality later in life. Probiotic strains have been developed as supplements for premature babies and to counter some of these ailments as well as to confer a range of health benefits. The ability to modulate the immune response and produce short-chain fatty acids, particularly acetate and butyrate, that strengthen the gut barrier and regulate the gut microbiome, makes *Bifidobacterium* a core component of a healthy infant through adulthood.

## 1. Introduction

As has been elegantly described, human milk has evolved to deliver all the nutrients, hormones, and bioactive compounds to give a newborn the best chance of surviving and thriving [1]. Within its complex composition lie oligosaccharides known to be utilized by the first organisms that colonize the gastrointestinal tract. Whether these compounds evolved to feed organisms, particularly bifidobacteria, or the organisms colonized to take advantage of them as nutrients, remains to be determined. Nevertheless, bifidobacteria are important bacteria for infants, and this mini review will explore their beneficial properties for early human life.

## 2. From Whence They Came

Acquisition of a healthy gut microbiota during the developmental stages of early human life plays a significant role in the health of that individual later in life. It has been proposed [2], though not universally accepted [3,4], that microbes begin to colonize the newborn while in the uterus. Then, at least during the natural birthing process, the organisms that live in the female genital tract have access to and interact with the baby. Gram-positive, polymorphic rod-shaped *Bifidobacterium* species are part of this maternal vaginal and fecal microbiota [5,6,7]. In the gut, *B. adolescentis*, *B. longum, B. angulatum, B. bifidum, B. pseudocatenulatum*, *B. breve*, *B. catenulatum, B. dentium*, and *B. pseudolongum* are commonly found [8]. To date, the role of each bacterial species acquired from those habitats in the infant’s development remains poorly understood, except for their decreased abundance in patients with atopic disease and intestinal ailments [9],. Apart from pathogens harming the infant, and organisms that are beneficial to infants early in development (for example, *Bacteroides thetaiotamicron* potentially aiding in intestinal cellular differentiation [10]) the bulk of research has been performed on *Lactobacillus* and *Bifidobacterium* species.

## 3. Why a Focus on *Bifidobacterium*?

The composition of the human gut microbiome is not static throughout development and undergoes dramatic changes as an individual grows older [11]. Highlighting this, the most dominant phyla in the adult gut microbiota include Actinobacteria, Proteobacteria, Firmicutes, and Bacteroidetes, with the latter two representing ~90% of the total; Actinobacteria (including bifidobacteria) are significantly less abundant [12,13,14]. This is in stark contrast to the microbial compositions observed throughout infancy, where *Bifidobacterium* spp. are drastically more abundant than in adults. In fact, the genus *Bifidobacterium* represents the most prominent microbial members in the gut of healthy, breast-fed infants [15,16,17]. This overrepresentation in the early gut environment suggests an important role in infantile development. As such, the origins, role, and potential therapeutic application of *Bifidobacterium* spp. in early human development, across multiple avenues, are discussed below.

That a strict and relatively fastidious anaerobe reaches the newborn gut and plays a key role in host health supports its co-evolution with humans [18]. In terms of overall abundance in the infant gut, bifidobacteria vary in terms of time, species, and strains. Part of the reason appears to be varied gene sets [19]; *Bifidobacterium* spp. demonstrate both inter- and intra-strain variance in metabolic and fermentative functions [20]. For example, genomic analysis has shown that individual strains of *B. longum* and *B. breve* vary in the number of human milk oligosaccharide (HMO) utilization genes, which alters their ability to use these compounds as a source of nutrients [21,22]. As such, the strains that can use HMOs efficiently are suspected to dominate the gut of a breast-fed infant. Changes in the diet and lifestyle of mothers in different parts of the world can affect the HMOs and bifidobacteria that colonize the infant’s gut. In a study performed on Bangladeshi infants, bifidobacterial dominance correlated with reduced colonization by organisms with antimicrobial resistance genes, thereby suggesting an important function for fighting infection [23]. A study on Malawian children showed higher proportions of bifidobacteria than in Finnish children, suggesting perhaps that diet impacts abundance [24], though this is difficult to pin down without a study examining confounding factors and dietary recall. As with any cause-and-effect study, large numbers of subjects would have to be included to ascertain direct correlations and interrogate mechanisms. Thus, the importance of one *Bifidobacterium* species over another, their abundance and metabolic activity, cannot easily be deciphered, though studies have shown different abilities to use fucosyllactose or sialyllactose [25]. Certainly, the use of antibiotics as well as the milk’s glycan composition are factors of importance [16]. 

A case has been made for a critical role of *B. longum* subsp. *infantis* due to its diverse genomic capacity and ability to digest and utilize HMOs [26]. If this is the case, it would make sense to supplement this species as a probiotic in infant formula. However, to date, single probiotic strains have been added to infant formula without any transparent reason. For example, formulas contain *Lacticaseibacillus* (formerly *Lactobacillus) rhamnosus* GG or *Bifidobacterium lactis* BB-12. The literature indicates these strains have very different attributes, yet the assumption of the parent and pediatrician is that they should confer the same health-promoting benefits. This puts into question which of these strains, if any, would lead to the best health outcomes for infants. Since few comparative studies have been done on two or more probiotic infant formulas, the question is difficult to answer. One clinical trial did show that a strain of *B. infantis* in either formula or human milk increased the fecal bifidobacterial numbers more so than *B. lactis* [27]. This study was not designed to establish a health benefit, but there was an assumption that an overall increase in *Bifidobacterium* species is desirable for infants. Further to other studies, this strain was commercialized and is now advertised as “the most infant-appropriate *B. infantis* strain”.

It is not the intent of this commentary to analyze the data supporting this statement, but it is worth asking the question of what evidence is required to select probiotic strains for universal usage in infants and in gauging one over another.

The desired health outcome of a probiotic is specific to the target disease and the strain(s) used. By ignoring differences that exist in function and metabolic capacity between bacterial strains, the unique effect of a probiotic on the host is ignored. Unfortunately, too little emphasis has been placed on strain properties for the desired application to humans. Many companies combine strains in an impromptu manner without considering the between-strain interference that could alter the desired outcome observed in clinical trials employing an individual strain [28,29]. With ethical issues surrounding the supplementation of live microorganisms to infants, such an intervention would require proof of the strain’s necessity in addition to rigorous safety testing. We have recently shown for *Lactobacillus crispatus* that metabolomic analysis can identify strains appropriate or not for probiotic applications to improve vaginal health [30]. It might not seem to be a relevant topic for a female infant but debilitating urinary tract infections can occur at that age [31]. Considering this is the stage where bacteria colonize the gut, implanting beneficial ones and reducing pathogens is important. Thus, the early-life application of probiotic strains, whether lactobacilli or bifidobacteria, requires an investigation of the properties and a rationale for their use, including their safety, not simply because they belong to those genera [28,29].

As an example, we recently examined four *Bifidobacterium* strains for their ability to counter the toxic effects of *p*-cresol, a compound detrimental to chronic kidney and cardiovascular health. It turned out there were some differences between *B. breve* HRVD521-US, *B. animalis* HRVD574-US, *B. longum* SD-BB536-JP and *B. longum* SD-CECT7347-SP (Unpublished data), though each appeared to have beneficial attributes. These experiments were performed in vitro and in a *Drosophila* model, which raises the question of how do you select strains and predict efficacy in humans? The answer is that models and genomic analysis can provide insight into the strain, but human studies alone can prove efficacy. Arguably, a strain can only perform tasks for which it has the genes. Whilst true, the environment within which it resides, in this case the gut, can alter gene expression and compound availability, and present molecules that the strain can then utilize. This is the case with *Clostridium* and *Enterobacteriaceae* spp. residing in the gut, which produce phenolic compounds including *p*-cresol from the metabolization of tyrosine and phenylalanine.

So, which properties are desirable for bifidobacteria in the infant gut and how can these influence the probiotic formulation that is being developed? We propose there are three important activities bifidobacteria carry out in the infant gut: they establish themselves as primary colonizers, allowing their health benefits to be ingrained; modulating immunological development; and producing metabolites that confer other physiological benefits.

## 4. Primary Colonization and Shaping Microbial Composition in the Gut

In the infantile gut environment, bifidobacteria engage in advantageous interactions with the host and other members of the microbiota that benefit intestinal and body-wide physiology. On the surface of bifidobacterial cells exist a myriad of proteins that facilitate their adhesion to intestinal epithelium [17,32,33]. This adherence is important as it can limit the colonization of pathogenic microbes by mitigating space and nutrient availability at the intestinal lining [17,32]. This process is enhanced by the presence of HMOs, which bifidobacteria use as growth promoters. The sequential establishment of a microbiota has been well characterized in the oral cavity, with primary then secondary colonizers taking on important roles [34]. Studies are required to gain this same insight into intestinal colonization [35].

To understand how the gut microbiota is established, a tremendous effort has been made to decipher the compositional shifts that occur throughout the first year of development. As the infant grows, the gut microbiota both increases and decreases in α-diversity and β-diversity, respectively, which is indicative of the increasing complexity of the community [15,36]. However, this is a non-random process driven by both environmental factors as well as the birthing method (i.e., vaginal vs. C-section) [15,37,38]. The first bacteria to colonize the gut are derived from vertical, mother–infant transmission [15]. Vaginally derived infants acquire bacterial communities from the vaginal and intestinal microbes of the mother, dominated by *Bifidobacterium*, *Lactobacillus,* and *Prevotella* [15,39], whereas C-section infants are more likely to be colonized by skin surface bacteria such as *Staphylococcus* and *Corynebacterium* [15,39]. Postnatal factors, the most important being breastfeeding, help shape the microbiota of children throughout the first year of life [15,40]. Notably, the gut microbiota develops more slowly than once thought, as evidenced by the functional and taxonomic difference between adult and child microbiotas [41]. Indeed, the gut microbiota composition of a 1-year-old is more similar to that of its mother than a newborn [15]. However, even after 12 months of life, *Bifidobacterium* and *Lactobacillus* dominate the intestinal environment of breast-fed infants; the overall abundance of *Bifidobacterium* is expected to decline, slowly but continuously, as one progresses through to adulthood [15,40,41,42]. While breast milk selects for these infant-associated genera, it is also breast-feeding, rather than the shift to solid foods, that is required for a successful transition to an adult-like microbiota, dominated by *Bacteroides* [12,13,14,15,40,42]. While the reason for this is not yet clear, the effect of breast feeding on the gut microbiota seems to extend into later stages of life and is tightly linked with bifidobacterial abundance in the intestinal environment.

A loss of bifidobacteria at an early age can cause a wide range of disorders. Specifically, a reduction in the abundance of the genus *Bifidobacterium* in infants has been shown to increase the prevalence of obesity, diabetes, metabolic disorder, and all-cause mortality later in life [17,43,44]. This might be because bifidobacteria are needed to increase the presence of other microbes associated with health. As a result of cross-feeding interactions, metabolites produced by bifidobacteria [45,46], including those formed from HMO utilization [47], select for the butyrogenic bacteria such as *Faecalibacterium prausnitzi, Anaerostipes,* and *Eubacterium* [48]. Butyrate is the main source of energy for colonocytes and is important for the maintenance of the epithelial barrier. The compound has also been shown to improve outcomes in colorectal cancers and metabolic diseases [49]. This offers a reasonable explanation for the reduced incidence of metabolic disease in individuals who were sufficiently colonized by bifidobacteria in early life. Furthermore, a loss of these important butyrate-producing microbes has been associated with conditions such as kidney stone disease and chronic kidney disease [48]; two conditions that are increasing in prevalence in children [50,51]. However, the list of important cross-feeding interactions, mediated by bifidobacteria, does not end here.

Other cross-feeding networks established between bifidobacteria and other commensals rely on the degradation of nutrients such as oligosaccharides, xylan, starch, arabinogalactan, mucin and more [52,53,54,55,56,57,58,59]. Importantly, the degradation of arabinogalactan establishes a network of *Bifidobacterium* and *Bacteroides* (a key member of the adult microbiota) that support one another by sharing catabolites [59]. These and other syntrophic interactions highlight the co-evolution of gut microbes and the human host. Many of these interactions seem to be mediated by bifidobacteria, which exemplifies their ecological role in obtaining and sharing substrates to and from other organisms [52,53,54,55,56,57,58,59]. Thus, bifidobacteria help establish and modulate microbiota composition and facilitate metabolic interaction to promote a healthy microbial community. Furthermore, the main fermentation metabolites of bifidobacteria, acetic and lactic acid, antagonize pathogens such as *Salmonella* and *Listeria* and can limit infection [15]. Taken together, these observations emphasize the importance of integrating bifidobacteria into the intestinal microbiota early in life.

Given the clear role that bifidobacteria play in establishing a healthy infant gut microbiota, and the transition to an “adult-like” composition, there is the potential to utilize certain strains to drive microbiome diversity. The most obvious group to benefit from probiotic supplementation are those delivered via C-section because they have much lower proportions of beneficial bacteria and take a longer time to develop a “normal” microbiota, compositionally speaking [60]. To date, some evidence exists to show that probiotic supplementation is sufficient to normalize the gut microbiota of C-section babies [61,62]. If this is true, then the early intervention of well-selected probiotic strains in these infants may provide a healthier start to development and prevent some of the chronic illnesses associated with microbial dysbiosis later in life [15,17,30,31,39,60]. However, investigations to elucidate which strain(s) are most effective remain to be conducted.

## 5. Impact of the Strains on the Host’s Immunity

The binding interaction between bifidobacteria and enterocytes plays a role in educating the immature immune system through the triggering of proinflammatory responses [33,63]. As mentioned, because C-section babies are exposed to fewer routes for the vertical transmission of microbes, the likelihood that they acquire microbes from the external environment instead of common anaerobes coming from the mother’s vagina or feces is increased [17,64]. Not surprisingly, the colonization of bifidobacteria in C-section babies occurs at a much slower rate compared to those born vaginally [65,66,67]. This delay could improve the adherence of potentially pathogenic microbes such as *E. coli* to the intestinal epithelium and could result in high titers of bacterial toxins in circulation or infection [68]. Depending on the bacteria present, this could increase the risk of disease in these individuals as they transition into adulthood [69].

Variations in the adherence ability between *Bifidobacterium* strains can cause immunological aberrations. For example, *B. adolescentis* is better at adhering to the intestinal lining than *B. bifidum*, and hence at utilizing nutrients found at this site and limiting pathogen burden [32,70]. Infants predominately colonized with *B*. *bifidum* rather than *B. adolescentis* are at greater risk of allergy [32,70]. Furthermore, the reduced colonization of *Bifidobacterium* is associated with a higher risk of other atopic diseases, including dermatitis and eczema. Considering that these are characterized by an overactive IgE immune response, it is likely that *Bifidobacterium* play a role in modulating the host’s response to common allergens. Unfortunately, the mechanisms behind how *Bifidobacterium* can regulate the immune system are not well known. Despite this, work is being done to elucidate the underlying causes for these observations, and one study showed that a reduction in *Bifidobacterium longum* prevents the maturation of circulating T-regulatory cells and increases the risk of allergy [71].

Multiple in vitro and animal studies have used a range of experimental protocols to predict how a strain will manipulate innate and adaptive immunity with limited success. Some successes have occurred when transferring the findings to humans, such as reducing allergic responses and inflammatory processes, including in infants [72,73]. Given that hosts may respond to certain strains and not others [74], and because *Bifidobacterium* strain propagation depends on which prebiotic it can assimilate [75], accurate predictions are difficult to achieve. Nevertheless, clinical studies have shown, for example, that bifidobacterial strains can improve plasma lipid profiles in children [76], and some can reduce the incidence of necrotizing enterocolitis in premature infants [77], although the extent to which immune modulation plays a role has not been defined. 

## 6. Bifidobacterial Metabolites

Beyond the surface-bound features that are beneficial to humans, bifidobacteria are also able to secrete factors that improve host health. Short-chain fatty acids (SCFAs) are the primary waste product of the microbiota that results from the fermentation of indigestible polysaccharides, including HMOs [78]. The most relevant SCFAs to human health are formate, acetate, butyrate, and propionate, because they account for the vast majority present in the colon [79]. These compounds are multi-functional in human health and play a significant role in gut barrier integrity, intestinal pH, and the inhibition of pathogens, but are of particular relevance to childhood development because they act as food for colonocytes [32]. By doing so, there is a reduction in the translocation of deleterious compounds such as lipopolysaccharides (LPS) and other bacterial toxins from the intestine into circulation, thereby protecting the infant [80]. Considering that LPS is present in baby formula and can increase the permeability of the infant’s intestinal epithelium, improving the gut integrity of infants not breastfeeding takes on even more significance [81]. The release of a broad range of SCFAs by bifidobacteria and their extrapolysaccharides utilized by other bacteria [82] also leads to a drop in pH, associated with enterocyte generation and improved colonic surface area, allowing more mineral absorption, which supports infantile development [83]. For example, negatively charged SCFAs conjugate with Ca^2+^ ions to improve passive diffusion through the lipid membrane of enterocytes. The significance of SCFAs is corroborated by the fact that a reduction in these compounds in the body is associated with many chronic diseases, including in the kidney [79,84,85]. However, it is not yet clear if these chronic conditions have origins in childhood.

As reviewed by Daisley et al. [86], acetate is emerging as a molecule that drives many important processes, including waste management, energy generation and the regulation of microbial communities. Recently, our group showed that acetate selects for beneficial *Akkermansia* in the colon [87]. This suggests that acetate is fundamental in the cross-feeding interactions between *Bifidobacterium* and butyrogenic bacteria. This is further supported by the fact that bifidobacterial-synthesized acetate is used to make butyrate directly [46,88,89]. Interestingly, although lactate can be used by some anaerobes to produce butyrate, it seems that acetate is still required in this process, further highlighting its importance.

While the focus of this review is not the role of acetate as a master regulator in the gut, the molecule has other beneficial properties relevant to infant well-being. Acetate can be formed from H_2_S and CO_2,_ and H_2_ by dissimilatory sulfate-reducing bacteria and acetogens, respectively [86,90,91]. While this represents a hyper-simplification of the complex underlying mechanisms of acetogenesis from intestinal gas, these processes have been described elsewhere [90,92]. Bloating caused by the over-production of gas in the colon can cause significant discomfort to an infant [93]. Therefore, acetate production might help provide relief.

Ultimately, the introduction of acetate-producing *Bifidobacterium* could select for a microbiota associated with good health. The fact that these organisms produce higher yields of acetate than other SCFAs further suggests an evolutionary contribution to infant health [94].

## 7. Further Potential

Two interesting areas of future potential applications of bifidobacteria are for brain and kidney health. Although heavily debated, there is growing evidence to suggest that microbes play a role in neurodevelopmental disorders such as autism. While work in animal models is not translatable to humans, a recent rodent model of autism indicated the resulting changes in social behavior correlated with alterations in bile acid and tryptophan metabolism [95]. One of the most significant findings from this study was a reduction in bifidobacterial colonization. As mentioned above, *Clostridium* and *Enterobacteriaceae* spp. residing in the gut produce *p*-cresol from the metabolism of tryptophan and the other aromatic amino acids tyrosine and phenylalanine. Increased levels of *p*-cresol exacerbate the autism-like behaviors of these rats, which has been corroborated in autistic children who have a higher burden of *p*-cresol in the urine, indicating greater systemic loads [96,97,98]. However, at the current time, it is not known whether the accumulation of *p*-cresol is the cause or result of autism spectrum disorder.

Chronic kidney disease management of children has improved but remains a major cause of reduced longevity [99]. Elevated *p*-cresol levels are also associated with chronic kidney and cardiovascular disease in adults. Of interest would be to examine the levels of these toxins in children, particularly those with kidney diseases, or indeed their mothers, since acute renal injury can arise in neonates and premature infants born with less than half the normal numbers of nephrons [100]. Recent work from our group has identified that four strains of bifidobacteria can sequester *p*-cresol from the extracellular environment and offer protection from the toxin in vivo (Unpublished data). As applications of *p*-cresol-sequestering probiotic bifidobacterial strains are safe for adults and children, it would be possible to see if this influences the incidence and management of children with autism spectrum disorder and kidney disease.

## 8. Conclusions

**Figure 1:**
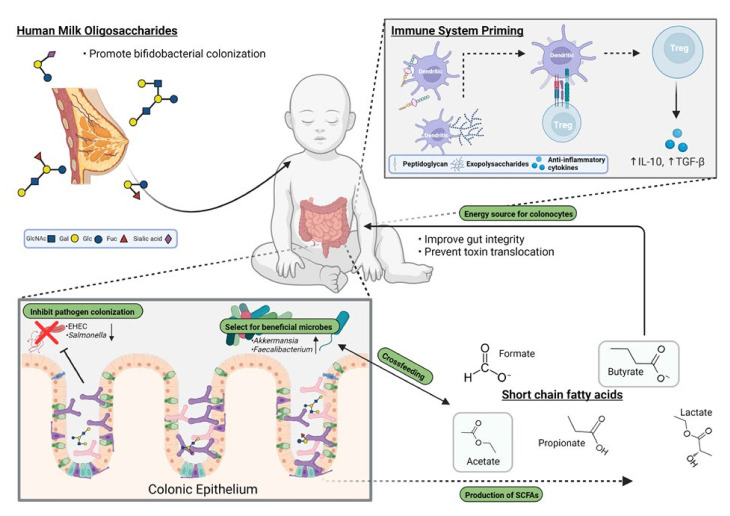
Influence of bifidobacteria on promoting a healthy gut microbiota and factors that affect their colonization.

In summary, *Bifidobacterium* species are important primary colonizers of the infant intestinal tract, and their abundance, especially following ingestion of HMOs, correlates with health (Figure 1). For premature babies, those delivered by C-section and those not gaining access to human milk, supplementation with probiotic strains is worthy of consideration, although more studies are required to select strains with appropriate properties. Much still needs to be done to correlate abundance, species, and function in healthy infants before selecting probiotic strains for infant formula. Ultimately, the risks associated with low bifidobacterial loads and potentially low levels of certain species could translate into diseases later in infancy through to adulthood.

## Data Availability

Not applicable.
